# *Cichorium intybus* L. Oligo-Polysaccharides (CIO) Exerts Antianxiety and Antidepressant Effects on Mice Experiencing Behavioral Despair and Chronic Unpredicted Mild Stress

**DOI:** 10.3390/foods14010135

**Published:** 2025-01-06

**Authors:** Yanqin Luo, Xueyi Bei, Yiwen Zhang, Xinran Sun, Yongzhi Zhao, Fang Chen, Ruile Pan, Qi Chang, Qinghu He, Xinmin Liu, Ning Jiang

**Affiliations:** 1 Research Center for Pharmacology and Toxicology, Institute of Medicinal Plant Development (IMPLAD), Chinese Academy of Medical Sciences and Peking Union Medical College, Beijing 100193, China; niqnayoul@163.com (Y.L.);; 2 Sino-Pakistan Center on Traditional Chinese Medicine, Hunan University of Medicine, Huaihua 418000, China; 3 Engineering Research Center of Storage and Processing of Xinjiang Characteristic Fruits and Vegetables, Ministry of Education, School of Food Science, Shihezi University, Shihezi 832000, China; 4 Institute of Drug Discovery Technology, Ningbo University, Ningbo 315211, China

**Keywords:** anxiety, behavioral despair, *Cichorium intybus* L. oligo-polysaccharides (CIOs), chronic unpredicted mild stress (CUMS), depression, mice, PI3K/AKT/mTOR

## Abstract

*Cichorium intybus* L. oligo-polysaccharides (CIOs), obtained from *Cichorium intybus* L., is a mixture of oligosaccharides and polysaccharides. This study explores the antianxiety and antidepressant effects and mechanisms of CIOs by using acute behavioral despair and chronic unpredictable mild stress mice models and measuring the levels of 5-HT and the expression of proteins related to the BDNF/ERK and PI3K/Akt/mTOR pathways. Moreover, 56 male C57BL/6N mice were used to test behavioral despair. They were randomized into seven groups (Control, Citalopram, CIO 12.5 mg/kg, CIO 25 mg/kg, CIO 100 mg/kg, and CIO 200 mg/kg) based on body weight; they were administered with the corresponding medication daily for 7 days; and behavioral tests were conducted on them (forced swimming test (FST) and tail suspension test (TST)) after 7 days. Seventy male C57BL/6N mice were adopted in the next part of the experiment and randomly divided into seven groups (Control, CUMS, Fluoxetine, MOO, CIO 25 mg/kg, and CIO 100 mg/kg) based on the sucrose preference index. Except for the control group, the other groups were subjected to 6 weeks of CUMS. From the fifth week of stress, the corresponding drugs were administered by gavage until the end of the behavioral tests. In the behavioral despair tests, the immobility time was significantly reduced in the FST and TST after the CIO (25 and 100 mg/kg) treatment of 7 days. After 6 weeks of chronic unpredicted mild stress (CUMS) treatment, CIO (25, 50, and 100 mg/kg) administration significantly reduced the number of buried beads in the marble burying test (MBT), decreased the latency in the novelty-suppressed feeding test (NSFT), and shortened the immobility time in the FST and TST. CIO administration significantly increased the sucrose preference index in the sucrose preference test (SPT). Additionally, CIO treatment increased hippocampal 5-HT levels while upregulating the expression of BDNF, P-PI3K/PI3K, P-ERK/ERK, P-Akt/Akt, and P-mTOR/mTOR. In summary, CIO exerted promising antidepressant effects in behavioral despair and antianxiety and antidepressant effects in CUMS-induced depressive mice. Moreover, CIO therapy was facilitated by increasing the 5-HT content, alleviating the damage of hippocampal neurons, and upregulating the BDNF/ERK and PI3K/AKT/mTOR cascade. Thus, CIO is a substance with the potential to treat anxiety and depression.

## 1. Introduction

Depression, a type of psychiatric disorder present worldwide, is characterized by emotional distress, distraction, and lack of interest, which is accompanied by sleep disorders, abnormal appetite, abnormal pain, and fatigue [1]. Currently, it has become an important public health problem [2]. Furthermore, depression has been one of the dysphrenial diseases with the highest disability rate since 2019 [3]. After the COVID-19 pandemic, many uncertainties arose, which would aggravate the development of depression [4,5]. It is expected that by 2030, depression will become the second leading cause of disability worldwide, which would cause a great number of economic losses [6,7,8]. There are some common antidepressants, such as selective serotonin reuptake inhibitors (SSRIs), noradrenergic reuptake inhibitors (NARIs), and serotonin and noradrenaline reuptake inhibitors (SNRIs), which are usually accompanied with serious side effects [9,10]. Therefore, it is urgent to find safer and more efficient agents against depression.

The pathogenesis of depression is complex and involves interactions at multiple levels. Research indicates that the mechanisms underlying depression are closely related to neurotransmitter dysregulation, neuroendocrine changes, neuroinflammatory responses, alterations in brain structure and function, and a deficiency of neurotrophic factors [11,12,13,14,15]. In particular, the monoamine neurotransmitter system, including serotonin, dopamine, and norepinephrine, is considered to be a core pathophysiological mechanism of depression due to its abnormal distribution and dysfunction [12]. Additionally, the excessive activation of the hypothalamic–pituitary–adrenal axis leads to elevated levels of glucocorticoids, as well as reduced secretion of the brain-derived neurotrophic factor (BDNF), which also play important roles in the onset of depression [13].

*Cichorium intybus* L., also called chicory, a perennial herb from the *Cichorium* genus, *Asteraceae* family, is an archeophyte of Mediterraneo–irano–turan origin with a long history of medicine which dates back to prehistorical times [16,17,18]. Recently, there is an increasing interest in chicory utilization for food production and supplementation. Certain substances found in chicory, including sesquiterpene lactones, polyphenols, inulin, and oligofructose, may be thought of as possible food functional carriers. Moreover, chicory has functions such as liver protection, lowering blood lipids, lowering blood sugar, lowering blood uric acid, anti-pathogenic microorganisms, antioxidant effects, and neuroprotective activities [19,20,21,22,23,24]. *Cichorium intybus* L. oligo-polysaccharides (CIOs) extracted from chicory, consist of 5–21 sugars equating to a content of 63.5%. Recent studies have found that CIO has potential antidepressant effects in mice, as evidenced by the forced swimming test (FST) and tail suspension test (TST) [25]. However, the antidepressant effects of CIO in chronically stressed animal models and the related mechanisms have not yet been investigated. Therefore, this study employs behavioral despair and CUMS models to explore the antidepressant effects and mechanisms of CIO in this study, providing new insights for the prevention and treatment of anxiety and depression.

## 2. Materials and Methods

### 2.1. Chemicals and Reagents

**Figure 1:**
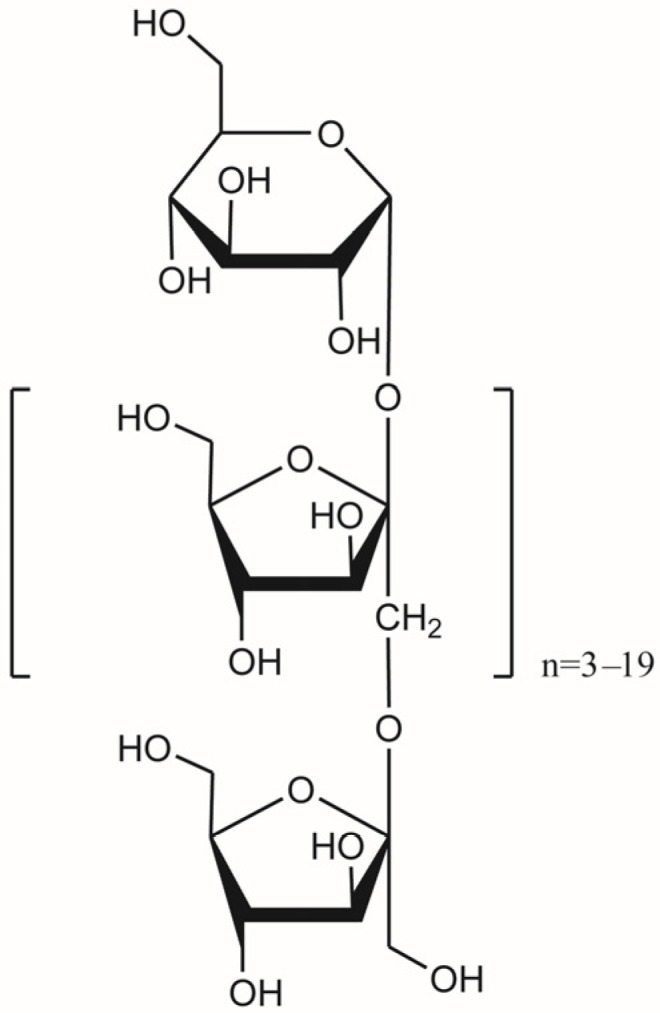
The structure of *Cichorium intybus* L. oligo-polysaccharides (CIO).

*Cichorium intybus* L. oligo-polysaccharides (CIO, purity of 75.25%) was provided by Tuolin Pharmaceutical (Qingdao) Co., Ltd. (Figure 1, Qingdao, China). Citalopram was acquired from Shanghai Macklin Biochemical Technology Co., Ltd. (Shanghai, China). Morinda oligosaccharides capsules were purchased from Beijing Tongrentang Co., Ltd. (Beijing, China), and fluoxetine was obtained from Changzhou Siyao Pharmaceutical Co., Ltd. (Changzhou, China).

### 2.2. Animals

A total of 126 male C57BL/6N mice (17~22 g), of SPF grade, were acquired from Hunan SJA Laboratory Animal Co., Ltd. (Changsha, China), and kept in a stable environment with a temperature of 23 ± 2 °C, humidity of 50 ± 5%, and a 12 h light/dark cycle. The experiment was carried out at the Hunan Prima Drug Research Center. All procedures were carried out in accordance with the NIH Guide for the Care and Use of Laboratory Animals and performed under the approval and supervision of the Ethical Committee of Hunan Drug Safety Evaluation (Approval No. SYXK 2020-0015).

### 2.3. Experimental Design

#### 2.3.1. Behavioral Despair Experiment

**Figure 2:**
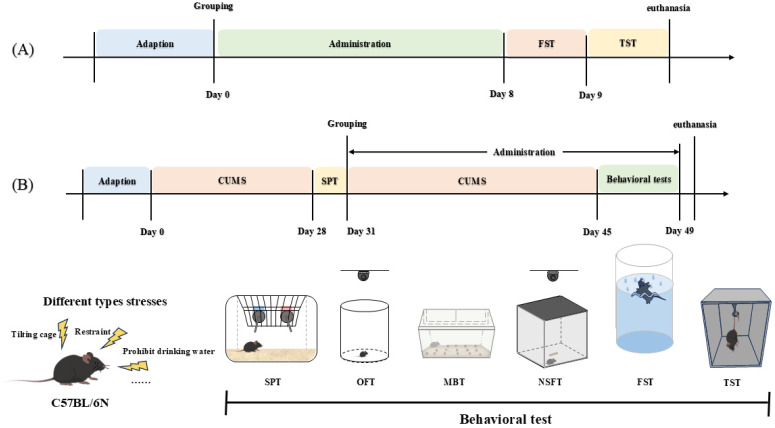
Experimental procedure. (**A**) Behavioral despair procedure. (**B**) Chronic unpredictable mild stress (CUMS) procedure. FST, forced swimming test; TST, tail suspension test; SPT, sucrose preference test; OFT, open field test; MBT, marble burying test; NSFT, novelty-suppressed feeding test.

To explore the optimal dosage of CIO against depression, 56 male C57BL/6N mice were selected for the behavioral despair experiment. Random allocation was based on weight after the adaptation period as follows: control; Cit (Citalopram, 10 mg/kg); CIO (12.5 mg/kg); CIO (25 mg/kg); CIO (50 mg/kg); CIO (100 mg/kg); and CIO (200 mg/kg). The control group was given pure water every day, and the other groups were given the corresponding concentrations of drugs. Animals were housed in standard mouse cages, with four mice per cage. After 7 days of continuous dosing, behavioral tests (forced swimming test and tail suspension test) regarding behavioral despair were conducted (Figure 2A).

#### 2.3.2. Chronic Unpredictable Mild Stress (CUMS) Experiment

This part of the experiment adopted 70 male C57BL/6N mice. The optimal dose of CIO was determined according to the results of the behavioral despair experiment. At the start of this experiment, ten mice were chosen at random, based on their weight, to serve as the control group. After being subjected to 28-day CUMS, using the sucrose preference index as a guide, the remaining mice were randomly assigned to the following 6 groups: CUMS; Fluoxetine (10 mg/kg); Morinda oligosaccharides (MOO, 50 mg/kg); CIO (25 mg/kg); CIO (50 mg/kg); and CIO (100 mg/kg). Apart from the control group, the other groups received matching medications; the control and CUMS groups received pure water daily; and the CUMS procedure was maintained. The medication was given once a day for 20 days, or until the behavioral testing was finished (Figure 2B). Animal husbandry was conducted in standard mouse cages, with four mice per cage for the control group, while the other groups were housed individually. The CUMS process would continue for 6 weeks in this part of the experiment. Following the behavioral tests, the mice were euthanized and their brains were removed. The hippocampus regions of the mouse brains were isolated and used for further analysis.

### 2.4. CUMS Stimulus Schedules

The CUMS procedures were strictly followed, as noted in Table 1.

### 2.5. Behavioral Tests

#### 2.5.1. Open Field Test (OFT)

The open field test was conducted according the official method, which was improved slightly compared to the previous one [26]. During the detection, the mice were placed in an activity box (40 cm × 40 cm × 35 cm, circle), and after adjusting to the environment for 3 min, the movement distance and time of mice within 10 min were recorded by the JL Behv animal behavior video analysis system 1.0 (Jiliang Software Technology Co., Ltd., Shanghai, China).

#### 2.5.2. Marble Burying Test (MBT)

The marble burying test was carried out as previously reported [27]. Twenty marble beads were placed in the cage with a cover to observe the burial process within 30 min, and the number of marble beads buried by mice was recorded after the experiment.

#### 2.5.3. Novelty-Suppressed Feeding Test (NSFT)

The novelty-suppressed feeding test was administered as previously reported [28]. Before the experiment began, the mice fasted for 24 h and could not refrain from drinking water. A food pill was positioned in the middle of an open box, while the animal was always placed in an identical direction and position. Feeding latency was observed and recorded from the time the animal was placed into the cage until the time it first picked up the food and bit down, lasting five minutes at a time.

#### 2.5.4. Sucrose Preference Test (SPT)

The sucrose preference test was conducted as previously described [29]. Three phases made up the experiment: the adaptation phase, the training phase, and the testing phase. The animals were given two bottles of 1% sucrose water during the adaptation phase so they could become used to drinking water containing sucrose for a whole day. During the training period, the animals received one bottle of 1% sucrose water and one bottle of pure water for the same amount of time. The animals were given a single bottle of 1% sucrose water and a single bottle of pure water for 24 h throughout the test period. They were also allowed to eat freely during the test; however, they went without water for 16 h before the test. During the test, the two bottles were exchanged to get around the location preference factor. The sugar water preference index in the testing phase was also calculated.

#### 2.5.5. Forced Swim Test (FST)

The forced swim test was carried out in a manner mentioned below. Simply, each mouse was made to swim for six minutes in a cylinder that was 20 cm high and 14 cm in diameter and contained 15 cm of water. The temperature of water is primarily maintained around 23 ± 1 °C [30]. The immobility time in the last four minutes were recorded by a software (JL Behv forced swimming video analysis system 1.0).

#### 2.5.6. Tail Suspension Test (TST)

Each mouse was wrapped with tape around its tail and then suspended on a tail-hanger [9]. The total experiment time was 6 min; the first two minutes were the adaptation period, and the last four minutes were the test period. The immobility time of mice during the test period was recorded by a video tracking software (Tail Suspension Real Time Analysis System 2.0).

### 2.6. Sample Collection

The hippocampus tissue was extracted the day following the behavioral tests, treated with liquid nitrogen, and kept in a refrigerator at −80 °C for further analysis.

### 2.7. Measurement of 5-Hydroxytryptamine Content in the Hippocampus

Hippocampus tissue was added to PBS (pH 7.4) according to the weight-to-volume ratio (1 g:9 mL), homogenized completely on ice, centrifuged at 3000 rpm at 4 °C for 20 min, and the supernatant was collected for an ELISA experiment. 5-HT concentration of the hippocampus was determined according to the instructions of a commercial kit (Jiancheng Biology, Nanjing, China).

### 2.8. Western Blotting Analysis

Following the behavioral test, half of the hippocampus tissue was homogenized on ice for 30 min after being added to the lysate in accordance with the percentage. Once the cleavage was finished, the supernatant was centrifuged for 30 min at 12,000 rpm and 4 °C. By utilizing the BCA protein quantification kit (Boopek Biotechnology Co., Ltd., Beijing, China), the protein concentration was ascertained following centrifugation. Protein samples were separated on a 10% polyacrylamide gel containing sodium dodecyl sulfate (SDS) and then moved to an NC membrane. After gently shaking the NC film in 5% skim milk, it was thoroughly cleaned three times using TBST. The NC film was incubated in the diluted primary antibody overnight at 4 °C, including GAPDH (1:4000, ABclonal), BDNF (1:1000, abcam), Akt (1:1000, CST), P-Akt (Ser 473, 1:1000, CST), ERK (1:1000, abcam), P-ERK (ERK1 phospho T202 + ERK2 phospho T185, 1:1000, abcam), mTOR (1:1000, CST), P-mTOR (Ser 2448, 1:1000, CST), PI3K (1:1000, CST), and P-PI3K (Tyr 458, 1:1000, CST), and then washed with TBST, while the HRP-conjugated secondary antibody was incubated slowly at room temperature. The protein bands were visualized by chemiluminescence (Amersham Pharmacia Biotech, Amersham, UK). Western blot images were obtained using the ImageJ analysis software (1.53k version, National Institutes of Health, Bethesda, MD, USA).

### 2.9. HE Staining

HE staining is a conventional staining method widely used in pathological diagnosis. The mice were anesthetized with ethyl carbamate, fixed with 4% paraformaldehyde, and the brain tissue was removed. After performing conventional paraffin embedding and coronal sectioning (5 μm), the sections were dewaxed, hydrated successively, stained by HE, dehydrated and sealed, and histological changes of mouse hippocampi were observed.

### 2.10. Statistical Analysis

All results are expressed as ± standard error of the mean (SEM). Data analysis was performed using the SPSS Statistics 26 software; GraphPad Prism 8.0.1 software was used for plotting; and ImageJ software was used to analyze protein bands. Data were statistically analyzed using one-way ANOVA. A *p*-value < 0.05 was considered statistically significant.

## 3. Results

### 3.1. Effect of Different Dosages of CIO on Depression

**Figure 3:**
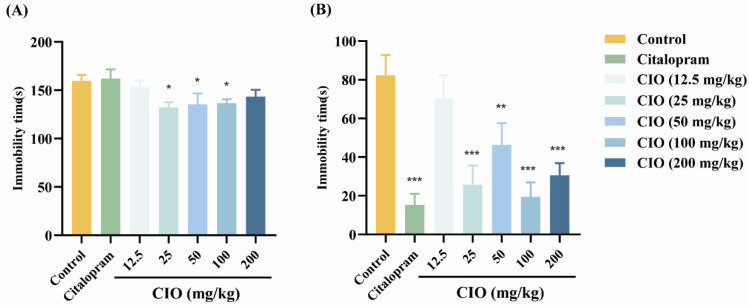
Effects of different dosages of CIO on depression. (**A**) FST; (**B**) TST. The data are expressed as mean ± SEM (*n* = 8). * *p* < 0.05, ** *p* < 0.01, *** *p* < 0.001; each group is compared with the control group.

After 7 days of dosage, CIO (25, 5,0 and 100 mg/kg) therapy markedly decreased the immobility time compared with the control mice in FST (Figure 3A, F [6, 49] = 2.657, *p* < 0.05). Compared with control mice, citalopram (10 mg/kg) and CIO (25, 50, 100, and 200 mg/kg) therapies significantly reduced the immobility time in TST (Figure 3B, F [6, 49] = 7.981, *p* < 0.01, *p* < 0.001).

### 3.2. Effects of CIO on CUMS-Induced Anxiety- and Depression-like Behavior in Mice

#### 3.2.1. Effects of CIO on Open Field Test

**Figure 4:**
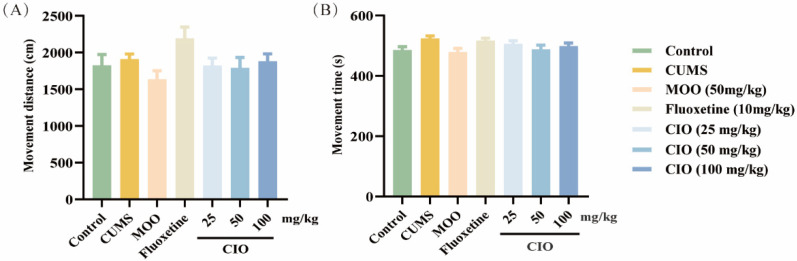
Effects of CIO administration on OFT. (**A**) Movement distance. (**B**) Movement time. The data are expressed as mean ± SEM (*n* = 10).

As shown in Figure 4, there were no differences in movement distance and time among all the groups of mice.

#### 3.2.2. Effects of CIO on the Marble Burying Test

**Figure 5:**
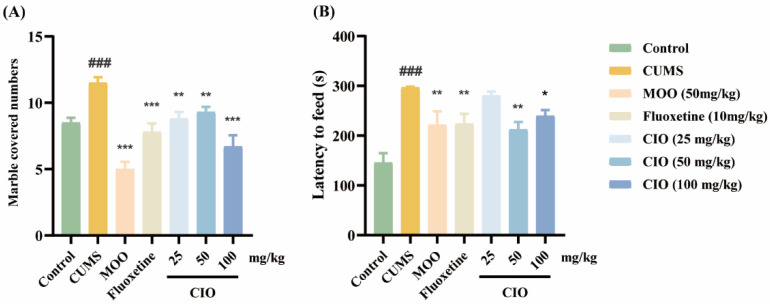
Effects of CIO administration on anxiety behavioral tests. (**A**) Marble burying numbers in MBT; (**B**) latency to feed in NSFT. The data are expressed as mean ± SEM (*n* = 10). ^###^ *p* < 0.001; each group was compared with the control group; * *p* < 0.05, ** *p* < 0.01, *** *p* < 0.001; each group was compared with the CUMS group.

As can be seen in Figure 5A, the number of marble beads buried in CUMS mice was significantly higher than that in control mice (F [6, 63] = 13.469, *p* < 0.001), while MOO (50 mg/kg), fluoxetine(10 mg/kg), and CIO (25, 50 and 100 mg/kg) treatments significantly reduced the amount of marble beads buried by mice (F [6, 63] = 13.469, *p* < 0.01, *p* < 0.001).

#### 3.2.3. Effects of CIO on the Novelty-Suppressed Feeding Test

As Figure 5B depicted, the feeding latency of CUMS mice was obviously longer than in control mice (F [6, 63] = 8.847, *p* < 0.001), while MOO (50 mg/kg), fluoxetine (10 mg/kg), and CIO (50 and 100 mg/kg) treatments significantly shortened the feeding latency compared with CUMS mice (F [6, 63] = 8.847, *p* < 0.05, *p* < 0.01).

#### 3.2.4. Effects of CIO on the Sucrose Preference Test

**Figure 6:**
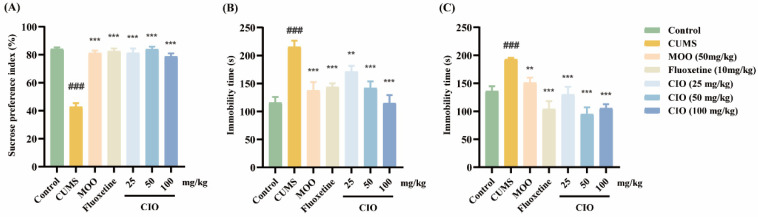
Effects of CIO administration on depression behavioral tests. (**A**) SPT; (**B**) immobility time in FST; (**C**) immobility time in TST. The data are expressed as mean ± SEM (*n* = 10). ^###^ *p* < 0.001; each group was compared with the control group; ** *p* < 0.01, *** *p* < 0.001; each group was compared with the CUMS group.

After a 6-week CUMS process, there was a remarkable decrease in the sucrose preference index compared with the control mice (Figure 6A, F [6, 63] = 46.953, *p* < 0.001). However, administration of MOO (50 mg/kg), fluoxetine (10 mg/kg), and CIO (25, 50 and 100 mg/kg) for 20 days significantly increased the sucrose preference index (Figure 6A, F [6, 63] = 46.953, *p* < 0.001).

#### 3.2.5. Effects of CIO on the Forced Swim Test

As Figure 6B shows, CUMS treatment dramatically increased the immobility time of mice (F [6, 63] = 9.047, *p* < 0.001); however, MOO (50 mg/kg), fluoxetine (10 mg/kg), and CIO (25, 50 and 100 mg/kg) therapies were able to shorten the immobility time in FST (F [6, 63] = 9.047, *p* < 0.01, *p* < 0.001).

#### 3.2.6. Effects of CIO on the Tail Suspension Test

The results shown in Figure 6C demonstrate that the CUMS treatment observably increased immobility time of mice (F [6, 63] = 11.223, *p* < 0.001); however, MOO (50 mg/kg), fluoxetine (10 mg/kg), and CIO (25, 50 and 100 mg/kg) administration could cut down the immobility time compared with CUMS mice (F [6, 63] = 11.223, *p* < 0.01, *p* < 0.001).

### 3.3. Effects of CIO on the 5-HT Level in the Hippocampus of CUMS Mice

**Figure 7:**
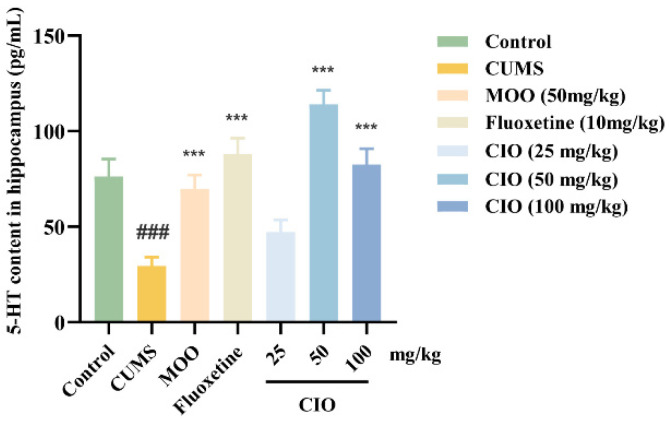
Effects of CIO on the 5-HT level in the hippocampus of CUMS mice. The data are expressed as mean ± SEM (*n* = 8). ^###^ *p* < 0.001; each group was compared with the control group; *** *p* < 0.001; each group was compared with the CUMS group.

We further studied the effects of CIO administration on the 5-HT level in the hippocampus of CUMS mice. As depicted in Figure 7, after the CUMS treatment, 5-HT content in the hippocampus significantly decreased compared with control mice (F [6, 63] = 13.703, *p* < 0.001). However, MOO (50 mg/kg), fluoxetine (10 mg/kg), and CIO (50 and 100 mg/kg) therapies observably enhanced the level of 5-HT in contrast to CUMS mice (F [6, 63] = 13.703, *p* < 0.001).

### 3.4. Effects of CIO on BDNF/ERK and PI3K/Akt/mTOR Signaling Pathways in the Hippocampi of CUMS Mice

**Figure 8:**
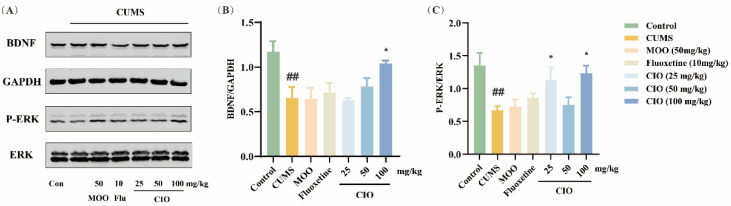
Effects of CIO on the expression levels of BDNF, ERK, P-ERK, and GAPDH in the hippocampi of CUMS mice. (**A**) Representative protein bands. (**B**) The ratios of BDNF/GAPDH. (**C**) The ratios of P-ERK/ERK. The data are expressed as mean ± SEM (*n* = 3–5). ^##^ *p* < 0.01; each group was compared with the control group; * *p* < 0.05; each group was compared with the CUMS group.

**Figure 9:**
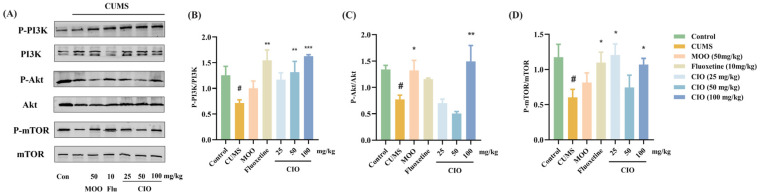
Effects of CIO on the expression levels of PI3K, P-PI3K, Akt, P-Akt, mTOR, and P-mTOR in the hippocampi of CUMS mice. (**A**) Representative protein bands. (**B**) The ratios of P-PI3K/PI3K. (**C**) The ratios of P-Akt/Akt. (**D**) The ratios of P-mTOR/mTOR. The data are expressed as mean ± SEM (*n* = 3–5). ^#^ *p* < 0.05; each group was compared with the control group; * *p* < 0.05, ** *p* < 0.01, *** *p* < 0.001; each group was compared with the CUMS group.

We further investigated the ways that CIO employs BDNF/ERK and PI3K/Akt/mTOR signaling pathways for producing its antidepressant effects. In the BDNF/ERK signaling pathway, in comparison with control mice, CUMS treatment significantly decreased the expression of BDNF/GAPDH in the hippocampi (Figure 8B, F [6, 27] = 4.620, *p* < 0.01). The expression of PERK/ERK was declined in the hippocampi as well (Figure 8C, F [6, 25] = 4.112, *p* < 0.01). However, CIO (100 mg/kg) treatment significantly increased the expression of BDNF/GAPDH (Figure 8B, F [6, 27] = 4.620, *p* < 0.05); moreover, CIO (25 and 100 mg/kg) treatment also clearly enhanced the expression of P-ERK/ERK (Figure 8C, F [6, 25] = 4.112, *p* < 0.05, *p* < 0.01). In the PI3K/Akt/mTOR signaling pathway, CUMS process significantly declined the expression of P-PI3K/PI3K, P-Akt/Akt, and P-mTOR/mTOR when compared with control mice (F [6, 16] = 3.602, *p* < 0.05, F [6, 15] = 4.911, *p* < 0.05, F [6, 21] = 2.249, *p* < 0.05, Figure 9B–D). On the other hand, fluoxetine (10 mg/kg) and CIO (50 and 100 mg/kg) administration markedly elevated the expression of P-PI3K/PI3K compared to CUMS mice (Figure 9B, F [6, 16] = 3.602, *p* < 0.01, *p* < 0.001). In the meantime, MOO (50 mg/kg) and CIO (100 mg/kg) therapies observably enhanced the expression of P-Akt/Akt (Figure 8C, F [6, 15] = 4.911, *p* < 0.05, *p* < 0.01), while fluoxetine (10 mg/kg) and CIO (25 and 100 mg/kg) treatments remarkably increased P-mTOR/mTOR (Figure 9D, F [6, 21] = 2.249, *p* < 0.05).

### 3.5. Effects of CIO on Hippocampal Neurons in CUMS Mice

**Figure 10:**
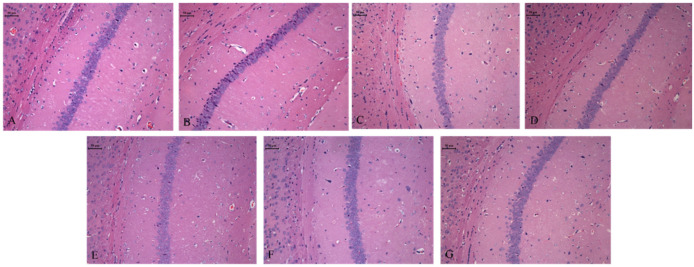
Effect of CIO on hippocampal histopathology in depressed mice (×200, CA1 section). (**A**) Control group. (**B**) CUMS group. (**C**) MOO group. (**D**) Fluoxetine group. (**E**) CIO (25 mg/kg) group. (**F**) CIO (50 mg/kg) group. (**G**) CIO (100 mg/kg) group.

As exhibited in Figure 10, the morphology of nerve cells in the control group is regular, with clear cytoplasm and uniform staining. Compared to the control group, the model group shows a reduced number of hippocampal nerve cells, with blurred cell contours, cytoplasmic loss, damaged nuclei, varying degrees of nuclear shrinkage, and deeper staining. However, after treatment with fluoxetine, MOO, and CIO, the nerve cells appear normal, with clearer cell contours, more distinct nuclear structures, and transparent cytoplasm.

### 3.6. Correlation Analysis Among Behavioral Tests, 5-HT Contents, and BDNF/ERK and PI3K/Akt/mTOR Signaling Pathways

**Figure 11:**
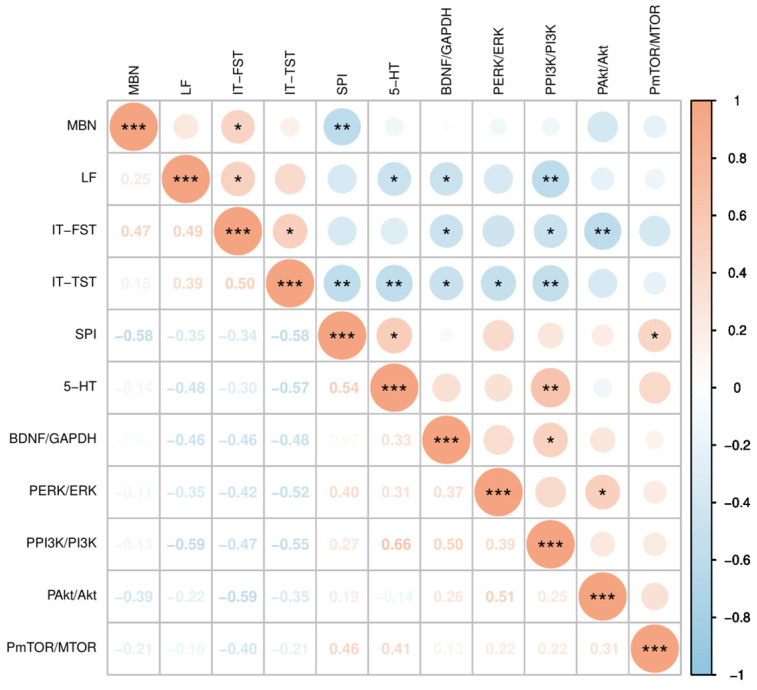
Correlation analysis among behavioral tests, 5-HT contents, and BDNF/ERK and PI3K/Akt/mTOR signaling pathways. The data are expressed as mean ± SEM (*n* = 21). MBN, marble burying numbers; LF, lantency to feed; IT-FST, immobility time in FST; IT-TST, immobility time in TST; SPI, sucrose preference index. Orange represents positive correlation (the darker the orange, the stronger the positive correlation); the blue represents negative correlation (the darker the blue, the stronger the negative correlation); * *p* < 0.05, ** *p* < 0.01, *** *p* < 0.001.

Pearson’s correlation coefficients were used for correlating behavioral indices, 5-HT content, and BDNF/ERK and PI3K/Akt/mTOR signaling pathways. As shown in Figure 11, the latency to feed (LF) in the novel suppression feeding experiment is negatively correlated with 5-HT levels, BDNF/GAPDH, and P-PI3K/PI3K (r = −0.48, *p* < 0.05; r = −0.46, *p* < 0.05; r = −0.59, *p* < 0.01); the immobility time (IT-FST) in the forced swimming test is negatively correlated with BDNF/GAPDH, P-PI3K/PI3K, and P-Akt/Akt (r = −0.46, *p* < 0.05; r = −0.47, *p* < 0.05; r = −0.59, *p* < 0.01); the immobility time (IT-TST) in the tail suspension test is negatively correlated with 5-HT levels, BDNF/GAPDH, P-ERK/ERK, and P-PI3K/PI3K (r = −0.57, *p* < 0.01; r = −0.48, *p* < 0.05; r = −0.52, *p* < 0.05; r = −0.55, *p* < 0.01); and the sucrose preference index in the sucrose preference test is positively correlated with 5-HT levels and P-mTOR/mTOR (r = 0.54, *p* < 0.05; r = 0.46, *p* < 0.05).

## 4. Discussion

In this study, we found that CIO treatment reduced the immobility time of depressed mice in TST and FST. CIO demonstrated the ability to improve anxiety-like and depression-like behaviors induced by CUMS in MBT, NSFT, SPT, FST, and TST. Additionally, our results suggest that CIO’s antidepressant activity may be attributed to its ability to enhance serotonergic neurotransmission, as evidenced by increased levels of 5-HT. Furthermore, CIO appears to exert neuroprotective effects, as indicated by the alleviation of the hippocampal neuron damage. These effects are potentially mediated through the modulation of the BDNF/ERK and PI3K/Akt/mTOR signaling pathways.

FST and TST are well-established methods, not only for detecting the extent of depression-like behavior in animals but also for evaluating the efficacy of potential antidepressant compounds [31,32]. In these tests, animals are placed in an inescapable, stressful environment, leading them to exhibit immobility, a behavior analogous to human depression that can be mitigated by antidepressant treatments [33]. We conducted behavioral despair tests to confirm the antidepressant effect of CIO and select the ideal dosage of CIO against depression. Our findings showed that CIO (25 and 100 mg/kg) treatment can shorten the immobility time of animals in the FST and TST. Based on these experimental results, CIO has an antidepressant effect on mouse models of behavioral despair.

It is well-established that CUMS is a classical model for studying depression [34]. Katz et al. developed this model in the early 1980s, based on the premise that prolonged exposure to an unsuitable and unpredictable environment can induce symptoms of stress, anxiety, and depression [35]. Thus, we utilized the CUMS to induce depression in this study. Subsequently, SPT, FST, and TST were chosen to evaluate the depression-like behavior of CUMS mice. In our study, CIO, MOO, and fluoxetine therapies significantly increased the sucrose preference index in SPT and shortened the immobility time in FST and TST. All the results in these behavioral tests indicate that CIO possesses the ability to mitigate the depression-like behavior caused by CUMS. Furthermore, given that approximately 85% of depression patients also experience anxiety [36], we included behavioral tests for anxiety (MBT and NSFT). Mice tend to engage in specific behaviors—such as digging, burying, grooming, and hoarding—when they are experiencing stress or anxiety; therefore, when the mice are housed with a marble, the animals start to bury it as a response. Mice buried more marbles in MBT, the more nervous they were [37]. NSFT is based on the conflict between the mice’s desire for food and the natural aversion for new environments [38]. Our study demonstrated that CIO, MOO, and fluoxetine administration significantly reduced the marble burying numbers in MBT. Moreover, CIO treatment can shorten feeding latency in NSFT. These results collectively suggest that CIO significantly alleviates depression and anxiety symptoms induced by CUMS, demonstrating its promising potential as an antidepressant agent.

The pathogenesis of depression is not yet fully understood, but the monoamine neurotransmitter hypothesis remains as one of the most prominent theories [39]. Extensive preclinical and clinical research has demonstrated that monoamine neurotransmitters, such as serotonin (5-HT), norepinephrine (NE), and dopamine (DA), play critical roles in the pathophysiology of depression [40,41]. Serotonin (5-HT), a monoamine neurotransmitter, is particularly important in the context of depression [42]. Research has shown that chronic stress can disrupt the 5-HT system, leading to depression-like symptoms [42]. Moreover, many antidepressant treatments, such as selective serotonin reuptake inhibitors (SSRIs), work by increasing 5-HT levels in the brain, which helps alleviate these symptoms [43]. In our study, CIO, MOO, and fluoxetine therapies significantly reversed the decrease in 5-HT levels caused by CUMS, further demonstrating the critical role of 5-HT in the pathophysiology of depression and suggesting that CIO has potential as an effective antidepressant.

**Figure 12:**
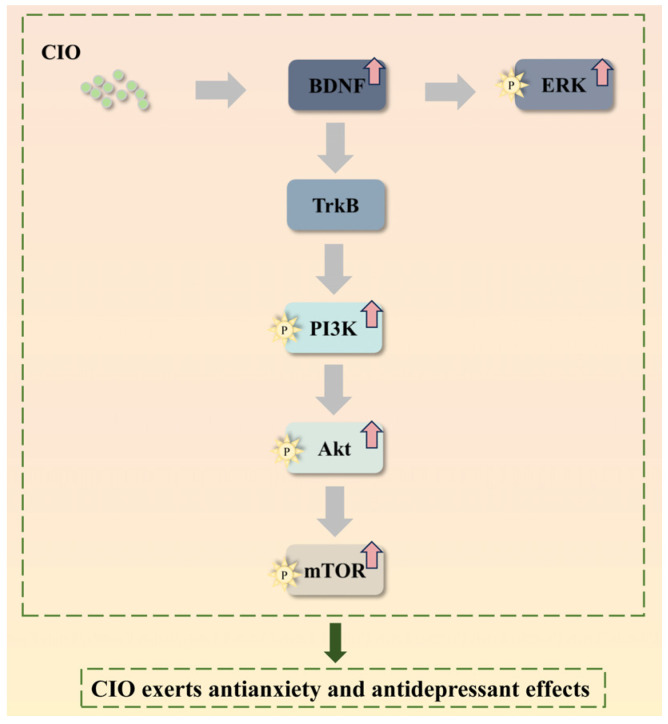
CIO exerts antianxiety and antidepressant effects via BDNF/ERK and PI3K/Akt/mTOR signaling pathways.

BDNF, which belongs to the neurotrophic factor protein family, is critically involved in depression [44]. It was reported that with the alleviation of the depression, the level of the BDNF would enhance as well [45]. ERK is implicated in depression as well [46], with numerous studies revealing that antidepressants have the capacity of increasing ERK in the hippocampus [47,48]. A decrease in BDNF expression leads to a subsequent reduction in ERK, as ERK is a downstream element of BDNF [49]. Conversely, the depression symptoms would be alleviated with increased ERK phosphorylation in mouse hippocampi following the BDNF treatment [50,51]. This study showed an increase in the expression of BDNF and ERK after the CIO treatment. Meanwhile, the PI3K/Akt/mTOR signaling pathway, downstream of the BDNF/TrkB, is considered vital in the treatment of depression [52]. CUMS has been shown to downregulate phosphorylated PI3K, Akt, and mTOR [53], which is able to be reversed by the fluoxetine treatment and thus exert antidepressant efficacy [54]. Previously, herb-like PPD [20(S)-protopanaxadiol] exerted antidepressant effects by activating the PI3K/Akt/mTOR pathway [9]. In agreement with previous studies [54], our results demonstrated that CUMS reduced BDNF/ERK and PI3K/Akt/mTOR signaling pathways in hippocampi of mice, whereas CIO treatment remarkably upregulated BDNF/ERK and PI3K/Akt/mTOR signaling pathways. These findings suggest that the antidepressant-like effects of CIO are mediated partially through the BDNF/ERK and PI3K/Akt/mTOR signaling pathways (Figure 12).

However, there are a few limitations that should be noted. Depression affects about 280 million individuals globally and accounts for 3.8% of the total population. Women are affected more than men by anxiety and depression according to WHO [55]. In our study, we only used male mice, and we will use both male and female mice in further research studies. Moreover, gut microbiota plays an important role in the improvement of anxiety and depression [56]. We do not know whether CIO plays an important role in the enhancement of gut microbiota in order to exert antianxiety and antidepression effects. In future research studies, we may focus on how CIO regulates gut microbiota to exert antianxiety and antidepressant effects.

## 5. Conclusions

In summary, the key findings in this study demonstrate that the treatment with CIO reversed anxiety- and depression-like behaviors induced by CUMS, as evidenced by improvements in SPT, MBT, NSFT, FST, and TST. Moreover, CIO therapy normalized the 5-HT content and hippocampal neuron integrity, while upregulating the protein expression of BDNF/ERK and PI3K/Akt/mTOR signaling pathways in the hippocampus. This study provides the first insight into the potential of CIO in the prevention of anxiety and depression.

## Figures and Tables

**Table 1:** CUMS stimulus schedules.

Time (Seven Days Round)	Stimulating Factors
Day 1	Restraining for 12 h and tilting the cage for 12 h.
Day 2	Fasting for 12 h, wet cage for 12 h, and the day and night were reversed.
Day 3	Ice water swimming (4 °C) for 5 min, strobe flashing for 12 h, and noise (85 dB) for 30 min.
Day 4	Water prohibition for 12 h, fasting for 12 h, and the day and night were reversed.
Day 5	Restraining for 6 h and tilting the cage for 12 h.
Day 6	Ice water swimming (4 °C) for 5 min, strobe flashing for 12 h, and noise (85 dB) for 30 min.
Day 7	Water prohibition for 12 h, strobe flashing for 12 h, and noise (85 dB) for 30 min.

## Data Availability

The data presented in this study are available on request from the corresponding author. The data are not publicly available due to privacy restrictions.

## Abbreviations

BDNF, brain-derived neurotrophic factor; CUMS, chronic unpredictable mild stress; CIO, *Cichorium intybus* L. oligo-polysaccharides; FST, forced swimming test; MBT, marble burying test; NSFT, novelty-suppressed feeding test; OFT, open field test; SPT, sucrose preference test; TST, tail suspension test
